# e-Cigarettes, Smoking Cessation, and Weight Change: Retrospective Secondary Analysis of the Evaluating the Efficacy of e-Cigarette Use for Smoking Cessation Trial

**DOI:** 10.2196/58260

**Published:** 2024-09-16

**Authors:** Lynnette Lyzwinski, Meichen Dong, Russell D Wolfinger, Kristian B Filion, Mark J Eisenberg

**Affiliations:** 1 Centre for Clinical Epidemiology Lady Davis Institute McGill University Montreal, QC Canada; 2 Department of Epidemiology, Biostatistics, and Occupational Health Faculty of Medicine McGill University Montreal, QC Canada; 3 JMP Statistical Discovery LLC Cary, NC United States; 4 Department of Medicine McGill University Montreal, QC Canada; 5 Division of Cardiology Jewish General Hospital Montreal, QC Canada

**Keywords:** nicotine, smoking cessation, e-cigarettes, vaping, weight change, weight gain

## Abstract

**Background:**

While smoking cessation has been linked to substantial weight gain, the potential influence of e-cigarettes on weight changes among individuals who use these devices to quit smoking is not fully understood.

**Objective:**

This study aims to reanalyze data from the Evaluating the Efficacy of e-Cigarette Use for Smoking Cessation (E3) trial to assess the causal effects of e-cigarette use on change in body weight.

**Methods:**

This is a secondary analysis of the E3 trial in which participants were randomized into 3 groups: nicotine e-cigarettes plus counseling, nonnicotine e-cigarettes plus counseling, and counseling alone. With adjustment for baseline variables and the follow-up smoking abstinence status, weight changes were compared between the groups from baseline to 12 weeks’ follow-up. Intention-to-treat and as-treated analyses were conducted using doubly robust estimation. Further causal analysis used 2 different propensity scoring methods to estimate causal regression curves for 4 smoking-related continuous variables. We evaluated 5 different subsets of data for each method. Selection bias was addressed, and missing data were imputed by the machine learning method extreme gradient boosting (XGBoost).

**Results:**

A total of 257 individuals with measured weight at week 12 (mean age: 52, SD 12 y; women: n=122, 47.5%) were included. Across the 3 treatment groups, of the 257 participants, 204 (79.4%) who continued to smoke had, on average, largely unchanged weight at 12 weeks, with comparable mean weight gain ranging from –0.24 kg to 0.33 kg, while 53 (20.6%) smoking-abstinent participants gained weight, with a mean weight gain ranging from 2.05 kg to 2.70 kg. After adjustment, our analyses showed that the 2 e-cigarette arms exhibited a mean gain of 0.56 kg versus the counseling alone arm. The causal regression curves analysis also showed no strong evidence supporting a causal relationship between weight gain and the 3 e-cigarette–related variables. e-Cigarettes have small and variable causal effects on weight gain associated with smoking cessation.

**Conclusions:**

In the E3 trial, e-cigarettes seemed to have minimal effects on mitigating the weight gain observed in individuals who smoke and subsequently quit at 3 months. However, given the modest sample size and the potential underuse of e-cigarettes among those randomized to the e-cigarette treatment arms, these results need to be replicated in large, adequately powered trials.

**Trial Registration:**

ClinicalTrials.gov NCT02417467; https://www.clinicaltrials.gov/study/NCT02417467

## Introduction

### Background

Cigarette smoking is a significant contributor to cardiovascular morbidity and mortality [1-4]. Although quitting cigarette smoking offers substantial cardiovascular benefits, the fear of gaining weight has been identified as a potential deterrent to smoking cessation [5,6]. Prevailing literature has shown that smoking cessation is associated with substantial weight gain, with a recent meta-analysis reporting a mean weight gain of 4.1 (95% CI 2.69-5.51) kg in individuals who quit [7] as well as an increase in BMI of 1.1 kg/m^2^ [7]. Similarly, studies have found an inverse relationship between BMI and smoking status compared with nonsmokers [8].

An overarching goal is to facilitate smoking cessation by providing safe alternatives to conventional cigarettes while minimizing unintended consequences such as the weight gain often experienced by quitters. According to the Global Burden of Disease study, 7.69 million deaths were attributable to smoking in 2019 on a global scale [9]. Furthermore, 200 million disability-adjusted life years were attributable to smoking in that same year [9]. Therefore, quitting smoking is especially important because it is associated with reduced risk of mortality, especially if done by the age of 40 years (90% reduction) [4]. However, weight gain in quitters has sometimes paradoxical effects on health and is linked with slightly reduced cardiovascular health benefits after smoking cessation [10] and increased risk of diabetes and obesity [11]. Obesity is a leading cardiovascular risk factor [12]. Thus, to truly lower cardiovascular risk among individuals who smoke, safe cessation methods are needed that also lower the risk of weight gain.

e-Cigarettes are increasingly being used for smoking cessation, and studies have demonstrated their efficacy for this purpose among adult smokers [13,14]. e-Cigarettes are considered safer alternatives than conventional cigarettes because they contain lower levels of harmful chemical toxins [15]. However, it should be emphasized that e-cigarettes are only recommended for adult smokers as a cessation harm reduction aid and not for minors among whom detrimental health effects have been documented [16].

Recent research further suggests that some individuals use e-cigarettes due to perceptions that they control weight gain, which may motivate them to transition from smoking cigarettes [17]. A systematic review found that vaping was prevalent in individuals who were overweight or obese [18]. e-Cigarettes containing nicotine may theoretically help control weight gain due to the direct effects of nicotine on metabolism through increased energy expenditure, suppressed appetite, and effects on adipose lipase levels [19-21]. In addition, the manner in which one holds an e-cigarette while making hand-to-mouth movement may simulate smoking behavioral patterns [22]. Replacing regular cigarettes containing tobacco with devices such as e-cigarettes that simulate this action may assist with maladaptive eating behavioral patterns that are often attributable to cigarette withdrawal [23]. However, there is limited evidence available regarding whether e-cigarettes may help with preventing the weight gain observed in smokers who quit.

### Objectives

This study aims to estimate the causal effect of e-cigarettes on smoking abstinence and weight changes among participants in the Evaluating the Efficacy of e-Cigarette Use for Smoking Cessation (E3) trial [8]. The E3 trial randomized individuals who smoke into 3 groups: nicotine e-cigarettes plus counseling, nonnicotine e-cigarettes plus counseling, and counseling alone. In our secondary analysis, we examine the relationship between e-cigarette use and weight gain by applying advanced causal inference methods while controlling for censoring and treatment selection biases. The rationale for applying advanced methods is largely due to the high loss to follow-up of participants during the COVID-19 pandemic and the fact that our manufacturer terminated the ongoing supply of e-cigarettes prematurely in the study. Therefore, we focus on weight change from baseline to 12 weeks, applying advanced novel statistical methods to account for missing data.

## Methods

We retrospectively analyzed data from the E3 trial, which was a multicenter study undertaken across Canada [24]. The trial details and methods have been previously described [24] and are briefly summarized in the E3 Trial subsection.

### Ethical Considerations

Ethics approval was received through the ethics review boards of each of the participating institutions across 17 centers in Canada. The research ethics board is the CIUSSS West-Central Montreal Board (Federalwide Assurance number 0796; project number MP-05-2015-322, 15-012). This study adhered to the international ethics regulations and the ethics principles outlined in the Declaration of Helsinki [25]. No additional ethics approval was needed because the original trial protocol included examining the relationship between e-cigarettes, weight change, and smoking abstinence.

### E3 Trial

Adults aged >18 years who smoked ≥10 cigarettes during the previous 12 months were eligible to participate in the E3 trial if they were motivated to quit. Inclusion in this substudy was restricted to participants who underwent weight measurements at 12 weeks’ follow-up. Details of the inclusion and recruitment criteria have been reported previously [24,26]. Briefly, participants must have smoked ≥10 cigarettes a day for a year and must have expressed a willingness to quit. The exclusion criteria included past use of a smoking cessation aid, past use of an e-cigarette over the previous 2 months or the use of an e-cigarette for a period of 1 week, and any history of a mental health problem or cancer. Individuals with a history of substance abuse were also excluded. Participants were recruited through advertisements in the media, including newspapers. Digital advertising was also part of the recruitment strategy, with advertisements placed on platforms such as Kijiji, Facebook, and Craigslist. Furthermore, patients were recruited directly from clinics, including smoking cessation clinics and walk-in clinics. The E3 trial team screened potential participants in person or via telephone interviews against the eligibility criteria [24].

Stratified randomization with block permutation (sizes 6 and 9; computer generated 1:1:1 sequence) was used to randomize participants to 1 of 3 groups: nicotine e-cigarettes (15 mg/ml) plus counseling, nonnicotine e-cigarettes (0 mg/ml) plus counseling, and counseling alone. The primary study treatment period was 12 weeks with a follow-up duration of 52 weeks. The primary end point of the E3 trial was point prevalence (7 days) of abstinence at 12 weeks, and the secondary outcomes included daily cigarette use and weight change (secondary analysis and outcome of interest). Participants were not prescribed a fixed daily dose and were instructed to use the e-cigarettes according to their personal needs and preferences (sessions and puffs varied according to habits and nicotine dependence). A schedule for nicotine tapering was not implemented, and participants were instructed to return both used and unused e-cigarettes cartridges at the end of the 12-week treatment period.

### Measures

Weight was measured in person with an eye-level physician scale at baseline and at follow-up clinic visits. Height was measured in person with a stadiometer. Table S1 in Multimedia Appendix 1 presents more details of the baseline variables. Participants were considered *abstinent* if they self-reported 0 cigarettes smoked in the past 7 days and recorded an expired carbon monoxide reading of ≤10 parts per million (ppm). If participants self-reported smoking cigarettes in the past week or an expired carbon monoxide reading of >10 ppm, they were identified as *nonabstinent* (or *returned to smoking*).

### Causal Statistical Analysis

We focused on weight change from baseline to 12 weeks, applying advanced novel statistical methods to account for missing data. Weight change at 12 weeks after randomization versus baseline was compared between the 3 randomized arms: nicotine e-cigarettes plus counseling, nonnicotine e-cigarettes plus counseling, and counseling alone. As bias can occur if weight and allocation group are associated with censoring, we used a doubly robust estimation (DRE) model for the main intention-to-treat (ITT) causal inference analysis [27]. The DRE model integrates a propensity score model and an outcome regression model that can help mitigate potential bias and increase the precision of causal effect estimates. While the ITT analysis addresses how weight changes with different interventions, it is also of interest to examine how weight changes when participants shift to e-cigarettes or stick to tobacco cigarettes. The raw data reveal a wide range of behaviors, from strict adherence to the treatment protocol to complete reversion to previous smoking habits. To better address the causal impact of e-cigarette use on weight change, we conducted several “as-treated” analyses. First, we used observed data to reconstruct the actual observed treatment arms (refer to the “Actual treatment” definition in Table S1 in Multimedia Appendix 1) and perform the same DRE analysis for these arms. Second, with access to 4 different continuous measures of e-cigarette use, we conducted an as-treated regression analysis using 2 different causal inference approaches: marginal structural model (MSM) [27] and generalized propensity score (GPS) [28]. Both use inverse probability weighting and make moderately different assumptions about the causal mechanism. We computed results for both methods because it was not clear from the study which of the methods was more appropriate given their assumptions. Furthermore, if both methods agreed, it would lend support to any causal claims made. The 4 continuous causal covariates we considered are as follows: *conventional cigarettes per week*, an abstinence measure where high values indicate that the person did not quit smoking and likely did not use e-cigarettes; *e-cigarette puffs used per week*, the product of the number of e-cigarette sessions per day, the number of e-cigarette puffs taken per session, and e-cigarette used days per week, which is arguably our best measure of e-cigarettes used per week (refer to the “e-Cigarette puffs used per week” definition in Table S1 in Multimedia Appendix 1); *used e-liquid cartridges returned*, a measure of how many e-cigarette cartridges were used and returned, which should be directly related to the degree of e-cigarette use; and *unused e-liquid cartridges returned*, a measure that inversely reflects e-cigarette use, with more unused cartridges indicating lower use.

Further sensitivity analysis involved the investigation of the overall effect of nicotine on weight change, where we performed a DRE analysis using a binary variable that indicated whether a participant was exposed to any type of nicotine-containing product during the 12 weeks of treatment.

All calculations were performed in JMP scripting language (JMP Statistical Discovery LLC). Extreme gradient boosting (XGBoost) [22] was used for missing data imputation. Missing value distribution, imputation details, and causal method (DRE, MSM, and GPS) details are presented in Table S2 in Multimedia Appendix 1, Multimedia Appendix 2, and Multimedia Appendix 3, respectively.

## Results

### Participant Characteristics

Overall, 376 participants were enrolled in the E3 study (Table S3 in Multimedia Appendix 1, Figure S1 in Multimedia Appendix 4), of whom 257 (68.4%) met all eligibility criteria for this substudy and were included in the analysis with the corresponding demographic, clinical, and smoking characteristics information (Table 1; Table S4 in Multimedia Appendix 1). More than four-fifths of the participants (225/257, 87.5%) self-identified as White. The participants’ mean age was 52 (SD 12) years. Of the 257 participants, 66 (25.7%) had respiratory disorders. Most of the participants (149/257, 58%) were heavy smokers, with a history of use of nearly 35 years, and had smoked a median of 365 packs per year in the past 10 years. Of the 257 participants, 204 (79.4%) had previously used abstinence aids, and on average, participants had attempted to quit 3 times (SD 3.6). The participants’ mean baseline weight was 81.3 kg. The nicotine e-cigarette plus counseling group (101/257, 39.3%) weighed slightly more than the nonnicotine e-cigarette plus counseling (91/257, 35.4%) and counseling alone (65/257, 25.3%) groups on average. Most of the participants (194/255, 76.1%) were overweight, and a substantial portion (95/255, 37.3%) were obese. The mean BMI at baseline was 29.4 kg/m^2^, and 37% (95/255) had a BMI of ≥30 kg/m^2^. Smoking behaviors and abstinence status per treatment assignment are summarized in Table 2.

**Table 1:** Baseline characteristics of participants by treatment group (n=257).

Characteristics				Nicotine e-cigarettes+counseling		Nonnicotine e-cigarettes+counseling		Counseling alone
**Demographic characteristics**								
	Age (y), mean (SD)		52 (13)		52 (12)		51 (11)	
	**Sex, n/N (%)**							
		Male	52/101 (51.5)		47/91 (51.6)		36/65 (55.4)	
		Female	49/101 (48.5)		44/91 (48.4)		29/65 (44.6)	
	**Self-reported race, n/N (%)**							
		Black	1/101 (1)		6/91 (6.6)		3/65 (4.6)	
		White	93/101 (92.1)		78/91 (85.7)		54/65 (83.1)	
		Other^a^	7/101 (6.9)		7/91 (7.7)		8/65 (12.3)	
	**Education, n/N (%)**							
		No degree, diploma, or certification	17/101 (16.8)		12/91 (13.2)		6/65 (9.2)	
		Completed secondary (high school)	22/101 (21.8)		25/91 (27.5)		13/65 (20)	
		Some college or university	44/101 (43.6)		35/91 (38.5)		30/65 (46.2)	
		Completed undergraduate degree or higher	18/101 (17.8)		19/91 (20.9)		16/65 (24.6)	
**Smoking characteristics**								
	Years smoked, mean (SD)		35 (14)		36 (14)		35 (13)	
	Cigarettes per day in the past 10 years, mean (SD)		21 (10)		22 (13)		21 (12)	
	Other smoker or smokers at home, n/N (%)		29/101 (28.7)		33/91 (36.3)		15/65 (23)	
**Anthropometrics**								
	Weight (kg), mean (SD)		83 (21)		82 (17)		80 (17)	
**Medical history^b^, n/N (%)**								
	Respiratory problems		22/101 (21.8)		29/91 (31.9)		15/65 (23.1)	
	High cholesterol levels		37/101 (36.6)		37/91 (40.7)		21/65 (32.3)	
	High blood pressure		32/101 (31.7)		34/91 (37.4)		15/65 (23.1)	
	Diabetes		12/101 (11.9)		21/91 (23.1)		10/65 (15.4)	
	History of heart disease		13/101 (12.9)		14/91 (15.4)		8/65 (12.3)	
	History of depression		34/101 (33.7)		33/91 (36.3)		21/65 (32.3)	
**Fagerström Test for Nicotine Dependence, n/N (%)**								
	Mild		14/101 (13.9)		22/91 (23.1)		14/64 (21.9)	
	Moderate		48/101 (47.5)		37/91 (40.7)		30/64 (46.9)	
	Severe		39/101 (38.6)		32/91 (35.2)		20/64 (31.3)	
**Beck Depression Inventory II, n/N (%)**								
	Minimal		66/100 (66)		65/91 (71.4)		39/64 (60.9)	
	Mild		17/100 (17)		13/91 (14.3)		12/64 (18.8)	
	Moderate		12/100 (12)		10/91 (11)		8/64 (12.5)	
	Severe		5/100 (5)		3/91 (3.3)		5/64 (7.8)	
Alcohol use per week, mean (SD)				3 (6)		4 (8)		4 (7)

^a^Participants were asked to select White, Black, or “Other, specify.” Self-reported “other” included Israeli, Indigenous, Asian, Pilipino, Urdu, Italian, Arab, Trinidadian, Moroccan, Nepalese, Spanish, Tunisian, and East Indian.

^b^Medical history was self-reported.

**Table 2:** Smoking behaviors by treatment assignment for uncensored participants at week 12 (n=257).

			Nicotine e-cigarettes+counseling (n=101)		Nonnicotine e-cigarettes+counseling (n=91)		Counseling alone (n=65)
Conventional cigarettes per week, mean (SD)			44 (51)		51 (57)		61 (57)
e-Cigarette puffs used per week, mean (SD)			262 (389)		97 (180)		—^a^
Used e-liquid cartridges returned, mean (SD)			13 (17)		5 (8)		—
Unused e-liquid cartridges returned, mean (SD)			15 (12)		22 (11)		—
**Smoking abstinence at week 12, n (%)**							
	Yes	25 (24.8)		19 (20.9)		9 (13.8)	
	No	76 (75.2)		72 (79.1)		56 (86.1)	

^a^Not applicable.

### Weight Change

**Figure 1:**
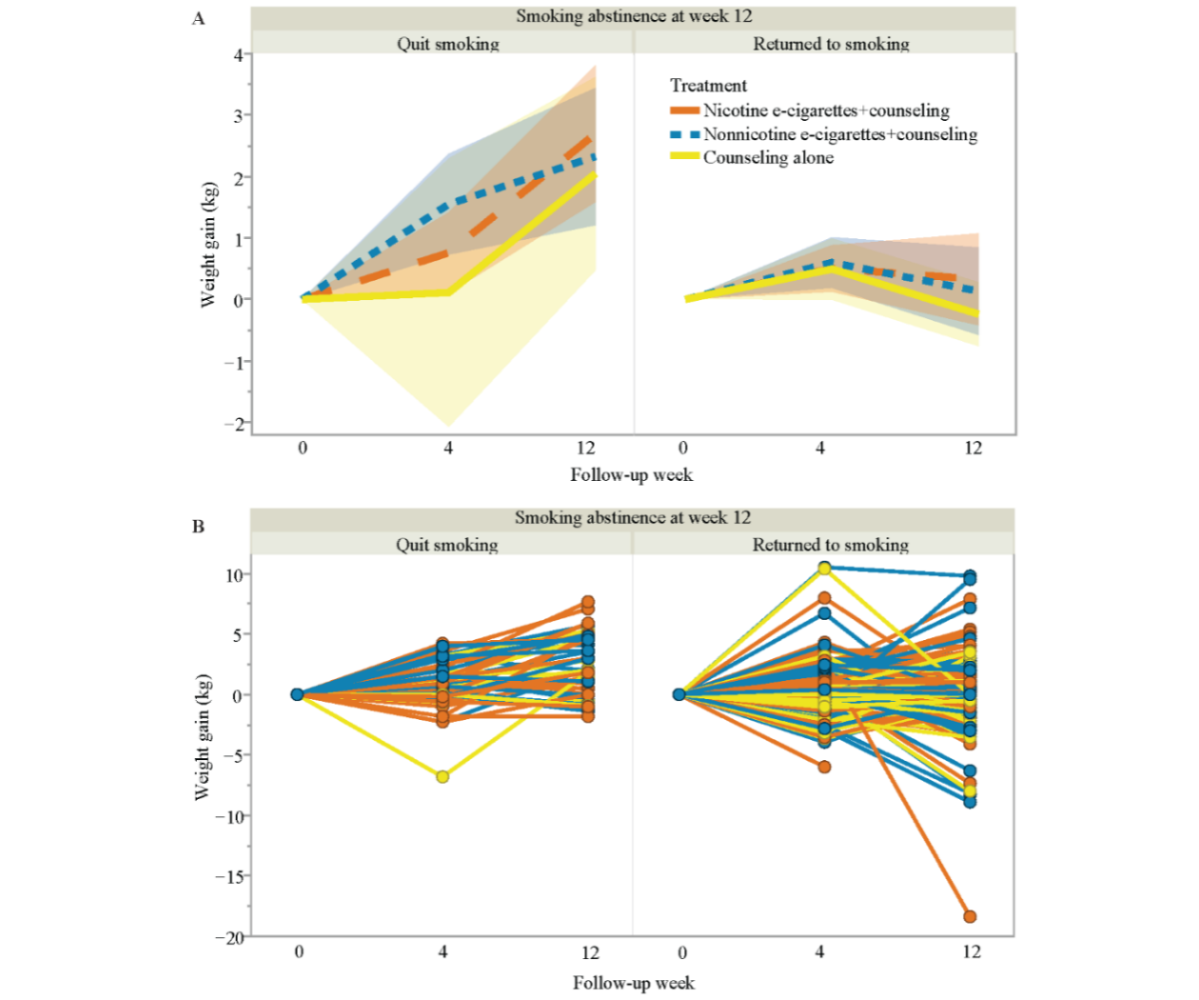
(A) Mean weight gain from baseline to week 12, shown by smoking abstinence at week 12 (quit smoking / returned to smoking) separately. Each error band is constructed using a 95% confidence interval. (B) Individual weight gain from baseline to week 12, shown by smoking abstinence at week 12 (quit smoking / returned to smoking) separately.

Figure 1A shows the crude mean weight change among participants who were deemed to have quit smoking and those who returned to smoking between baseline and 12 weeks’ follow-up within their assigned groups. The mean weight gain for participants who returned to smoking in the nicotine e-cigarettes plus counseling group (76/101, 75.2%) was 0.33 (95% CI –0.42 to 1.07) kg, whereas among those who quit smoking in the same group (25/101, 24.8%), the mean weight gain was 2.70 (95% CI 1.59-3.81) kg. In the nonnicotine e-cigarettes plus counseling group, participants who returned to smoking (72/91, 79%) showed a mean weight gain of 0.13 (95% CI –0.58 to 0.84) kg, while for those who quit smoking in the same group (19/91, 21%), the mean weight gain was 2.32 (95% CI 1.21-3.44) kg. In the counseling alone group, participants who returned to smoking (56/65, 86%) exhibited a mean weight change of –0.24 (95% CI –0.76 to 0.28) kg, whereas participants who quit smoking in the same group (9/65, 14%) had a mean weight gain of 2.05 (95% CI 0.48-3.62) kg. Figure 1B shows the individual trajectories for weight gain along the follow-up weeks by smoking abstinence status at week 12. It indicates that the weight gain at the follow-up visits varied substantially, regardless of smoking abstinence status. Specifically, the weight of individuals who resumed smoking remained relatively stable, with changes fluctuating around 0 kg, while those who quit smoking experienced an overall increase in weight.

### Causal Statistical Analysis

**Figure 2:**
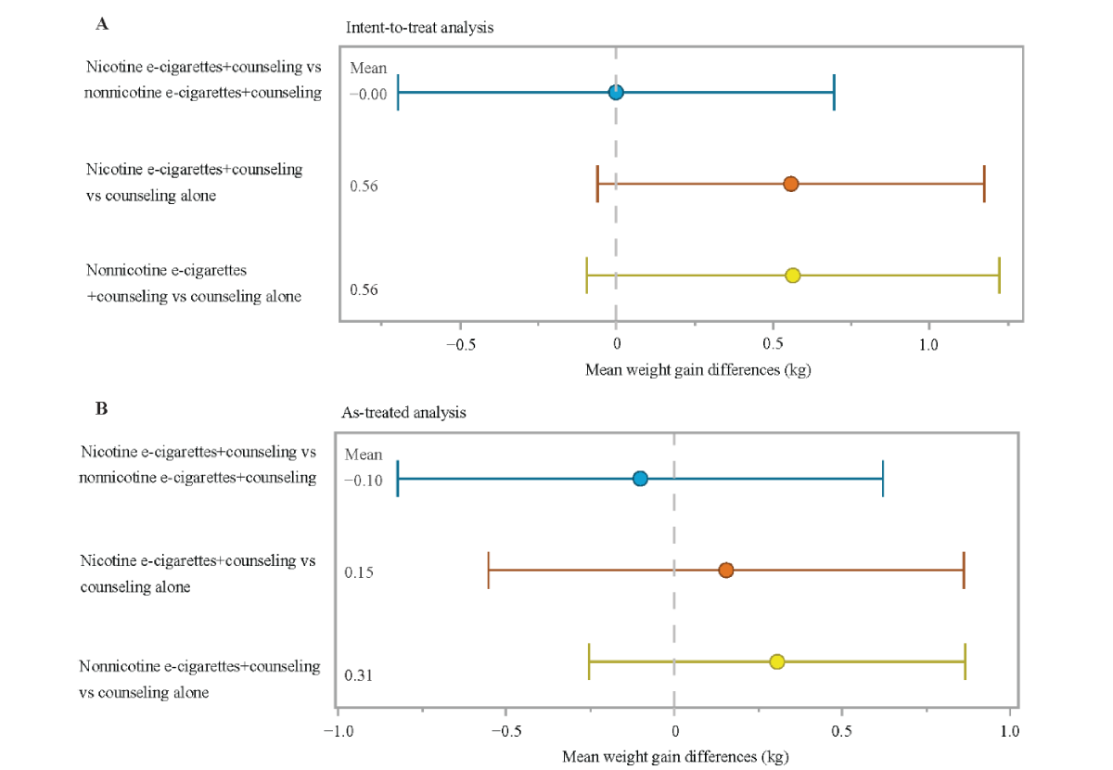
Pairwise comparisons with 95% confidence intervals between the three arms from a DRE model for intention-to-treat (A) and as-treated (B) analysis. In both models, censoring selection bias is adjusted. Treatment selection bias is also adjusted in the as-treated analysis. In (A) the intervention variable is the randomized arms; in (B) the intervention variable is the actual treatment arms (See Supplement table for the details of the intervention definitions). Note* Baseline variables used in the IPW analysis include: age, gender, baseline weight, height, education, average cigarettes per day smoked in the past 10 years, years smoked, Fagerström score, BDI score, Other smoker at home, high cholesterol, history depression, HBP, respiratory problems, history heart disease, diabetes, alcohol use, smoking abstinence at week 12.

Three pairwise comparisons with 95% CIs are shown for the ITT analysis (Figure 2A) and the as-treated analysis (Figure 2B). Refer to Table S1 in Multimedia Appendix 1 for definitions of the interventions. Although none of the comparisons were statistically significant at the 5% level, the nicotine e-cigarettes plus counseling group exhibited a mean weight gain of 0.56 (95% CI –0.08 to 1.13) kg versus the counseling alone arm, whereas the nonnicotine e-cigarettes plus counseling group had a mean weight gain of 0.56 (95% CI –0.14 to 1.23) kg versus the counseling alone arm. It is possible that contamination bias may have impacted the findings because some control patients were exposed to forms of nicotine from different sources outside of the trial. Thus, we undertook analyses for any nicotine use, including all types of nicotine substitution aids (eg, chewing gum and lozenges). In the DRE analysis for any nicotine use (data not shown), we found a mean difference of –0.18 (95% CI –1.12 to 0.64) kg between the participants who used nicotine and those who did not use nicotine. With the CI containing 0, the result indicates that there is no clear evidence of a causal relationship between nicotine exposure and weight gain.

**Figure 3:**
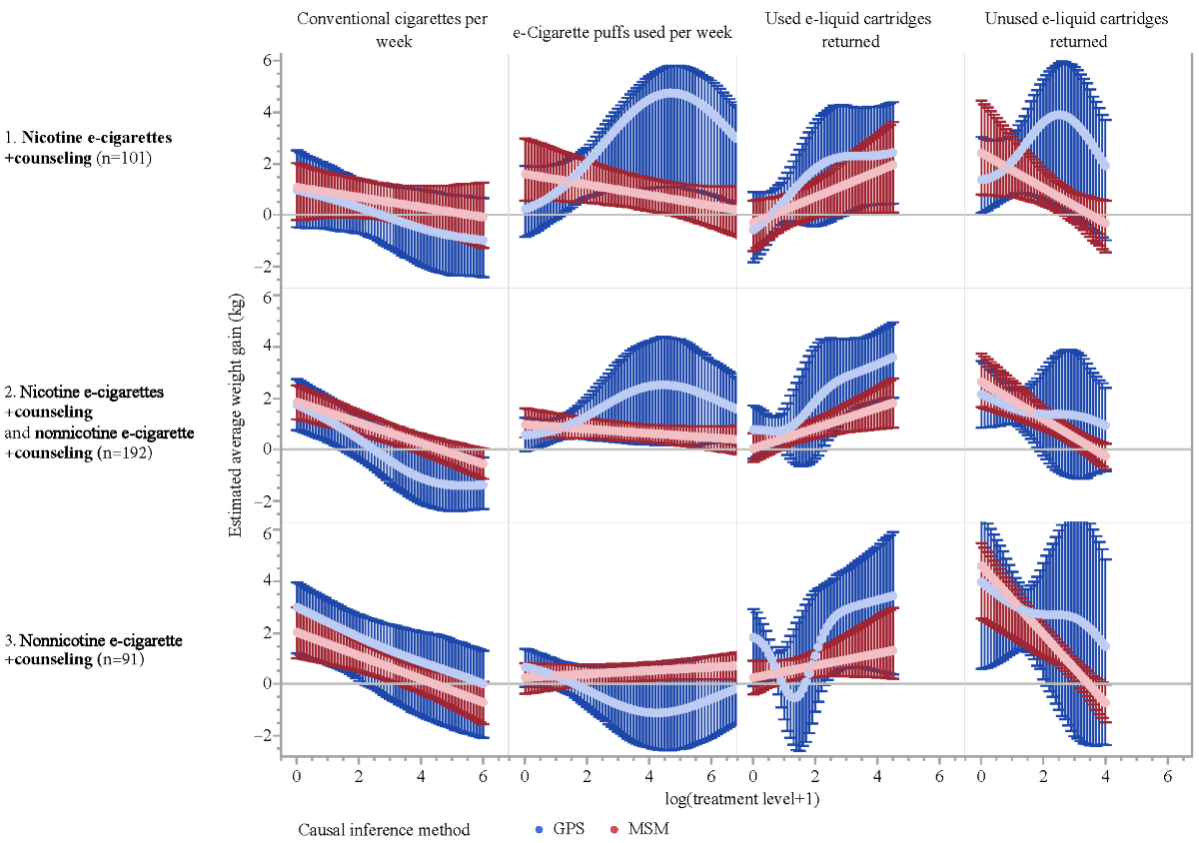
Results of As-Treated Causal Regression Analysis. Causal regression curves with bootstrap confidence limits are shown in a 3 x 4 grid, corresponding to five different subsets of data and four different causal covariates (log(1+x) transformed). For x-axis, 0 = log(0+1) represents the level of 0 on the original scale; similarly, 2, 4 and 6 represent the level of 6, 54, and 402 respectively on the original scale. The red and blue curves correspond to IPW with MSM and GPS causal inference methods, respectively. The columns correspond to (i) Conventional cigarettes per week; (ii) E-cigarette puffs used per week; (iii) Used e-liquid cartridges returned; (iv) Unused e-liquid cartridges returned. The analyses were performed for five subsets of data separately, as labeled on the right Y-axis, where participants are from one or two of the randomized treatment groups, with rows in the grid corresponding to (i) nicotine e-cigarettes plus counseling; (ii) nicotine e-cigarettes plus counseling and nonnicotine e-cigarettes plus counseling; (iii) nonnicotine e-cigarettes plus counseling. Note * Any of the curves in Figure 3 can be used to roughly answer “what-if” questions by finding a value of interest on the x-axis and projecting through the curve to the y-axis. GPS: generalized propensity score; MSM: marginal structural model.

Figure 3 displays a grid of causal estimation curves from our as-treated causal analyses with 4 continuous smoking-related variables (columns) in 3 subset analyses (rows). The first column of curves indicates that conventional cigarette use was negatively correlated with weight gain, as expected, in all 3 subsets, with consistent results with the GPS (blue) and MSM (red). The second column of curves suggests that the results for *e-cigarette puffs per week* disagree somewhat between GPS and MSM, although the CIs mostly overlap. The MSM curves (red) are linear and appear similar to those for conventional cigarettes, with less weight gain predicted for higher values. The GPS curves (blue) are nonlinear and show an opposite effect for lower values and positive correlation with weight gain, with peak gains of 2 to 5 kg at approximately 53.6 puffs per week (calculated as *e*^4^ – 1). The disagreement between the GPS and MSM results stems from disparate assumptions of the methods. This discrepancy (the second, the third, and the fourth column in Figure 3) leads to inconclusive findings, with no strong evidence supporting a causal relationship between weight gain and the 3 e-cigarette–related variables.

It is interesting to note that the MSM slopes (red curves in the third column) for *used e-liquid cartridges returned* go in opposite directions with the slopes for conventional cigarette use, indicating a potential positive correlation between weight gain and e-cigarette use. Furthermore, the downward slopes of the MSM curves (red in the fourth column) for *unused e-liquid cartridges returned* corroborate this finding. The MSM curves hint that nicotine e-cigarettes plus counseling could mitigate weight gain in at least some individuals (third column, first and second rows, in Figure 3).

Overall, the results from the as-treated analyses are largely inconclusive and consistent with the main ITT analysis results. Our extensive as-treated analysis does confirm the negative correlation between the use of conventional cigarettes and weight gain, lending some credence to other estimates obtained using the same methods.

## Discussion

### Principal Findings

This secondary analysis of the E3 trial was designed to investigate the evidence base for the use of e-cigarettes for controlling weight gain in smokers who quit smoking. e-Cigarettes have been previously shown to be effective in controlling cravings and are relatively safe for adult smokers who transition to them [29]. Our overarching aim was to further determine whether e-cigarettes may also mitigate the weight gain observed in smokers who quit smoking cigarettes.

We found that both nicotine and nonnicotine e-cigarettes have little impact on preventing smoking cessation–associated weight gain. From the crude analysis, we found that there was a positive average weight gain in participants who were smoking abstinent, regardless of treatment group, compared with those who returned to smoking (Figure 1). Adjusted for baseline variables, treatment selection bias, and censoring bias, both ITT and as-treated analyses (Figures 2 and 3) suggest that there is little statistical evidence supporting the hypothesis that nicotine or nonnicotine e-cigarettes helped prevent weight gain associated with smoking cessation during the 12-week treatment period. However, our extensive analysis with the continuous smoking-related variables not only confirms the finding that the more conventional cigarettes participants use, the less likely they are gain weight, but also sheds light on quantifying the relationship between weight gain and e-cigarette measures. It is promising to further understand the relationship between e-cigarette use and weight gain due to smoking cessation by applying our approach using more precisely measured e-cigarette use variables.

Our previously published study found a median weight gain of 4.8 kg in quitters [5], consistent with findings in the literature indicating an average weight gain of approximately 5.0 kg at 6 months [30]. Hence, the crude average weight gain in this study seems to be lower than what has been reported in the literature. While this study used a more rigorous analytical approach, it is worth noting that the most significant weight gain often occurs over a full year of cessation [31]. Therefore, it is likely that greater weight gain would have been observed at a longer period of follow-up. In addition, there was a slightly smaller proportion of participants (56/121, 46.1%) in the counseling alone control arm who did not return for a follow-up, and this may have partially impacted the overall mean weight gain.

A past prospective intervention in smokers who quit and switched to e-cigarettes also did not see significant effects of e-cigarettes on weight at the 3-month time point [32]. However, the authors found that at a longer period of follow-up, specifically at week 52, e-cigarettes mitigated the effects of abstinence-related weight gain [32]. Likewise, another study also found that individuals who quit smoking and switched to e-cigarettes had minimal weight gain at the 12-month follow-up [33]. Thus, there is a possibility that had this study been undertaken for a longer duration, then similar effects could have been found. This highlights that there is a need to undertake further research for a period of at least 1 year. While a recent systematic review found some evidence of e-cigarettes for controlling weight gain, it was not supported in in vitro studies compared with in vivo studies [18].

We considered the impact of seasonal weather changes on weight in Canada, noting higher enrollment in the early months (January and February). Some research also indicates that individuals are more likely to gain weight during the cold winter season as a result of consuming more calorie-dense comfort food and performing less exercise [34]. While such participants may be more likely to gain weight than those who were randomized during the spring, this would need to be associated with either (1) randomized treatment (which it was not) to bias the results by treatment group or (2) smoking status to bias the results of the analyses of weight gain by treatment status. While theoretically possible, it is unlikely, and if an association was present, it would likely have only a modest impact on our results.

It may also be worthwhile to note that our study population was slightly more overweight than most populations of smokers who tend to weigh less than nonsmokers, given the negative relationship between smoking and body weight gain [35]. Obesity prevalence was 37% (37/101) for the nicotine e-cigarettes plus counseling group, 40% (36/90) for the nonnicotine e-cigarettes plus counseling group, and 34% (22/64) for the counseling alone group, exceeding the reported rates in some literature.

### Clinical and Public Health Implications

Currently, there is a need for more rigorous randomized controlled trials followed over a longer period to gain more research evidence on the relationship between e-cigarette use and smoking outcomes before recommendations can be made. However, addressing weight loss in smokers who quit is crucial. Without interventions to prevent weight gain, adult smokers may feel reluctant to quit [36,37], while quitters face risks of diabetes and obesity from rapid and significant weight gain [38]. Clinically significant weight gain, defined as an increase of ≥5% in body weight, needs to be prevented because it increases the risk of metabolic syndrome [39]. This remains a difficult public health problem that requires urgent attention from primary care physicians and public health practitioners. Minimizing risks in smoking and quitting is warranted. In addition, previous studies found that 60% of the participants returned to smoking at 1 year, highlighting the need to focus on long-term smoking cessation efforts [6]. A recent study combined nicotine patches and e-cigarettes with nicotine and found that the combination was the most effective approach [40], which provides interesting insights for potentially studying long-term abstinence in the future. Besides ensuring sustained smoking abstinence, there is a need to limit cardiometabolic risk factors stemming from weight gain and endothelial dysfunction. Quitters who gain weight also have elevated blood pressure [5]. Reducing weight gain risk may be an important motivator for patients, and clinicians must look at the long-term clinical picture of sustained abstinence and weight control. To truly lower the cardiovascular risk in patients, smoking cessation cannot happen in isolation without targeted weight loss and weight gain prevention interventions because both obesity and smoking cigarettes are leading cardiovascular disease risk factors [41].

Currently, lifestyle interventions consisting of increasing physical activity [42] and modifying dietary intake [43,44] are effective for weight control. Quitters may benefit from a dual program that provides them with (1) safe cigarette replacement aids and (2) diet and physical activity interventions. There is a need for more discussion on weight gain prevention during smoking cessation consultations between patients and their physicians.

### Limitations

Our study has several potential limitations. First, while we applied the advanced causal inference methods to our analyses, our study is limited by a small sample size, and this has important implications given our null findings. It should be stressed that the counseling alone control group only had a minority of participants (9/65, 14%) who abstained from smoking at week 12. Approximate post hoc power calculations for the 3 pairs of comparisons indicate that our analyses were underpowered. To achieve the approximate 80% power to detect the estimated differences, sample sizes would have needed to be increased by a factor of 2 to 3. Details of the post hoc power calculations are provided in Multimedia Appendix 5. In a future research study, we could perform a meta-analysis that pools results from trials testing similar interventions. We would also call for studies with extended follow-up to definitively assess the effect of e-cigarettes on weight changes.

Second, like the vast majority of smoking cessation trials, the E3 trial used point prevalence abstinence as the primary end point [24,26]. However, the *continuous abstinence rate* could also be considered because it more accurately reflects sustained smoking cessation and may be closely linked to assessing postcessation weight changes over time (Multimedia Appendix 6). However, while the continuous abstinence rate captures sustained changes, because the E3 trial did not have a “hard” quit date, and participants were allowed to taper, many participants would have been considered nonabstinent immediately at the start of follow-up; for example, there were only a few participants (9/65, 14%) in the control group who abstained continuously at 12 weeks. The resulting small sample size makes it more restricted with this approach, and the results should be interpreted with caution and cannot be used alone to ascertain the full effects. Therefore, we consider the 7-day point prevalence more clinically important than continuous abstinence.

Third, we also performed a logistic regression investigating the potential baseline factors that affect attrition (Table S5 in Multimedia Appendix 1). Attrition bias, which is a form of selection bias, may have potentially impacted the results for a certain subset of participants. The fact that fewer individuals with heart disease (35/67, 52%) returned during the follow-up period may suggest that they were more likely to experience health-related barriers or complications that contributed to dropping out of the study. In addition, it is possible that the effects of e-cigarettes on weight gain could vary in racially diverse groups and younger age groups—the majority of the participants (155/257, 60.3%) were middle aged (aged 45-65 years)—although the distributions of characteristics were similar across the treatment groups. It is also worthwhile to consider self-selection bias stemming from the enrollment of participants into the study. As is frequently the case in many clinical trials, individuals self-selected to participate in the trial, and this may theoretically result in some selection bias, for example, individuals who were more enthusiastic about using e-cigarettes or who were more educated may have been more likely to participate, and this may have potentially impacted generalizability. While we used rigorous statistical methods to minimize potential bias, some residual bias is still possible.

Fourth, although we have a rich set of variables to adjust for, we could not adjust for all confounders in our analysis. The 3 e-cigarette–related continuous variables involved in the more targeted causal analysis are the best available variables to reflect the actual e-cigarette use, but the accuracy may be compromised due to potential inaccuracies in self-reporting, unsupervised use, and the possibility of partial cartridge returns.

Fifth, the E3 study used only 1 type of e-cigarette, which was specifically created for use in trials. As the participants may have different personal preferences, using a single e-cigarette option may have affected adherence to the intervention. This may limit the generalizability of the findings, given that they are specific to the e-cigarette brand that we used in our study.

Sixth, while the smokers had smoked 20 cigarettes a day, they did not obtain an equivalent dose of nicotine from vaping; smoking 20 cigarettes a day would be roughly equivalent to 200 to 300 puffs per day, compared with 37 puffs per day in the nicotine arm and 14 puffs per day in the nonnicotine e-cigarette arm. In addition, we used self-reported abstinence measurements and expired carbon monoxide levels to determine smoking cessation, which may introduce subjective reporting bias and have inherent limitations [45]. Setting an expired carbon monoxide threshold of <10 ppm might have overestimated the intervention’s impact on smoking cessation because higher thresholds are associated with an increased likelihood of demonstrating abstinence [46].

Finally, a limitation of this study is the potential underuse of the intervention (ie, e-cigarette use) offered to evaluate its impact on weight gain associated with smoking cessation. This disparity in nicotine dose and the level of ritualistic substitution may have nullified any potential effect of e-cigarette use on postcessation weight gain. The study’s conclusions might have differed if the ritualistic behavior and nicotine intake from vaping were comparable to those of tobacco cigarette smoking.

### Conclusions

In summary, e-cigarettes seem to have minimal effects on mitigating the weight gain observed in individuals who smoke and subsequently quit at 3 months. However, given the modest sample size of the E3 trial and potential underuse of e-cigarettes among those randomized to the e-cigarette treatment arms, these results need to be replicated in large, adequately powered trials. Future studies could make weight gain the primary target and take advantage of more straightforward causal inference methods available from randomized controlled trials. Such studies should aim to enhance participant retention, minimize concurrent use of abstinence aids in the control arms, follow participants for longer periods of time, and adjust for as many potential confounders as possible.

## Appendix

Multimedia Appendix 1 Supplementary Tables.

Multimedia Appendix 2 Imputation details.

Multimedia Appendix 3 Causal Statistical Modeling Details.

Multimedia Appendix 4 Supplementary Figure.

Multimedia Appendix 5 Power Analysis.

Multimedia Appendix 6 Continuous Abstinence Rate (CAR) analysis.

## Abbreviations

DRE: doubly robust estimation
E3: Evaluating the Efficacy of e-Cigarette Use for Smoking Cessation
GPS: generalized propensity score
ITT: intention-to-treat
MSM: marginal structural model
PPM: parts per million
XGBoost: extreme gradient boosting

## Data Availability

The data sets generated and analyzed during this study are available from the corresponding author on reasonable request. The code is available from the corresponding author on reasonable request.
