# Supplementary material for: e-Cigarettes, Smoking Cessation, and Weight Change: Retrospective Secondary Analysis of the Evaluating the Efficacy of e-Cigarette Use for Smoking Cessation Trial
Source: JMIR Public Health Surveill. 2024 Sep 16;10:e58260. doi: 10.2196/58260 (PMC11443201; doi:10.2196/58260)
Supplement: Multimedia Appendix 1 [file publichealth_v10i1e58260_app1.docx]

**Multimedia Appendix 1. Supplementary Tables**

**Table S1. Definitions of key variables.**

| **Variable** | **Definition** |
| --- | --- |
| **Smoking abstinence** | =1 if Carbon Monoxide <10 and conventional cigarettes used days=0. Missing values were set to 0, which means that the subject returned to smoking at that time point. |
| **E-cigarette puffs used per week** | is the product of number of e-cigarettes sessions per day, number of e-cigarettes puffs taken per session, and e-cigarettes used days per week. If “other e-cigarettes sessions” information is available, add the product of other e-cigarettes sessions, number of other e-cigarettes puffs taken per session, and other e-cigarettes used days per week to the above number. This variable represents the number of total puffs they used the e-cigarette per week, regardless of the source of e-cigarettes. |
| **Any smoking cessation aid** | If used any of the following, the value is 1 (YES): Varenicline, Bupropion, NicotinePatch, NicotineGum, NicotineInhaler, NicotineLozenge, NicotineQuickMist, Other e-cigarettes, Other aids. |
| **Actual treatment** | = “Nicotine e-cigarettes” if any of the following bullets is satisfied:   - Randomized Arm = nicotine e-cigarettes AND (Number of e-cigarettes sessions per day >0 or Number of e-cigarettes puffs taken per session >0 or E-cigarettes used days per week >0 or Used e-liquid cartridges returned >0) - Other e-cigarettes used days per week >0 AND Other e-cigarettes contain nicotine =1   = “Nonnicotine e-cigarettes” if any of the following bullets is satisfied:   - Randomized Arm = nonnicotine e-cigarettes AND (Number of e-cigarettes sessions per day >0 or Number of e-cigarettes puffs taken per session >0 or E-cigarettes used days per week >0 or Used e-liquid cartridges returned >0) - Other e-cigarettes used days per week >0 AND Other e-cigarettes contain nicotine =0   = “Counseling alone”, if no indication of e-cigarettes usage. |
| **AnyNicotineUse** | If any of the following bullets is satisfied the value will be 1(YES).   - NicotinePatch/NicotineGum/NicotineInhaler/NicotineLozenge/NicotineQuickMist=1 - Conventional cigarettes per week >0 - Carbon Monoxide>=10 - Other e-cigarettes contain nicotine =1 - Actual treatment = “Nicotine e-cigarettes” |
| **Years smoked conventional cigarettes** | the difference between age and age at first regular smoking. |
| **Alcohol use per week** | was derived from alcohol use per week or alcohol use per month variables. |
| **Education** | includes four levels: No degree, diploma, or certification; Completed secondary (high school); Some college/university; Completed undergraduate degree or higher. |
| **Fagerström Test for Nicotine Dependence score** | is categorized into three levels: mild (0-3), moderate (4-6), severe (>=7). |
| **Beck Depression Inventory II score** | is categorized into four levels: minimal (0-13), mild (14-19), moderate (20-28), severe (>=29). |
| **Interventions** | For ITT analysis, the interventions are the three randomized arms: Nicotine E-Cigarettes + Counseling, NonNicotine E-Cigarettes + Counseling, and Counseling alone.  For As-Treated analysis with three arms (Figure 2), the interventions are defined as the “**Actual treatment**” variable above.  For the As-Treated analysis with the continuous variables (Figure 3), the interventions refer to the amount of E-Cigarettes or Conventional cigarettes consumed, represented by the four variables. |

**Table S2. Missing counts distribution for the baseline variables for 257 patients in the main analysis.**

| **Variable Name** | **Nicotine e-cigarettes plus counseling** | **Nonnicotine e-cigarettes plus counseling** | **Counseling alone** |
| --- | --- | --- | --- |
| Age | 0 | 0 | 0 |
| Alcohol use per week | 0 | 0 | 0 |
| Cigarettes/day in the past 10 years | 0 | 0 | 0 |
| Beck Depression Inventory II | 1 | 0 | 1 |
| Diabetes | 0 | 0 | 0 |
| Education | 0 | 0 | 0 |
| Fagerström Test for Nicotine Dependence | 0 | 0 | 1 |
| Sex | 0 | 0 | 0 |
| High Blood Pressure | 0 | 0 | 0 |
| Height | 0 | 1 | 0 |
| High Cholesterol | 0 | 0 | 0 |
| History Depression | 0 | 0 | 0 |
| History Heart Disease | 0 | 0 | 0 |
| Other smoker(s) at home | 0 | 0 | 0 |
| Respiratory Problems | 0 | 0 | 0 |
| Smoking Abstinence at week 12 | 0 | 0 | 0 |
| Weight Baseline | 0 | 0 | 0 |
| Weight Gain (kg) | 0 | 0 | 0 |
| Years Smoked | 0 | 0 | 0 |

**Table S3. Missing weight measurement distribution by visit time.**

| **Missing weight measurement** | **Baseline** | **4 weeks** | **12 weeks** | **24 weeks** | **Total** |
| --- | --- | --- | --- | --- | --- |
| No | 375 | 293 | 257 | 219 | 1144 |
| Yes | 1 | 60 | 68 | 93 | 222 |
| Total | 376 | 353 | 325 | 312 | 1366 |

**Table S4. Demographic distribution for all 376 subjects**

|  | | | Nicotine  E-Cigarettes  + Counseling  (N = 128) | Nonnicotine  E-Cigarettes  +Counseling  (N = 127) | Counseling  Alone  (N = 121) |
| --- | --- | --- | --- | --- | --- |
| Demographic Characteristics | | |  |  |  |
|  | Age – mean years (SD) | | 53 (13) | 52 (13) | 53 (12) |
|  | Sex | |  |  |  |
|  |  | Male – n (%) | 63 (49) | 71 (56) | 64 (53) |
|  |  | Female – n (%) | 65 (51) | 56 (44) | 57 (47) |
|  | Self-Reported Race | |  |  |  |
|  |  | White – n (%) | 120 (94) | 111 (87) | 104 (86) |
|  |  | Black – n (%) | 1 (1) | 7 (6) | 3 (2) |
|  |  | Other^a^ – n (%) | 7 (6) | 9 (7) | 14 (12) |
|  | Education | |  |  |  |
|  |  | No degree, diploma, or certification – n (%) | 23 (18) | 15 (12) | 15 (12) |
|  |  | Completed secondary (high school) – n (%) | 25 (20) | 33 (26) | 32 (26) |
|  |  | Some college/university – n (%) | 58 (45) | 55 (43) | 47 (39) |
|  |  | Completed undergraduate degree or higher – n (%) | 22 (17) | 24 (19) | 27 (22) |
| Smoking Characteristics | | |  |  |  |
|  | Years smoked – mean (SD) | | 35 (14) | 35 (14) | 35 (13) |
|  | Cigarettes/day in the past 10 years – mean (SD) | | 21 (11) | 22 (12) | 22 (11) |
|  | Other smoker(s) at home – n (%) | | 40 (31) | 45 (35) | 36 (30) |
| Anthropometrics (N complete follow-up*) | | |  |  |  |
|  | Weight – Mean kg (SD) | | 81 (20) | 80 (17) | 81 (20) |
| Medical History^c^ | | |  |  |  |
|  | Respiratory problems – n (%) | | 31 (24) | 40 (32) | 34 (28) |
|  | High Cholesterol – n (%) | | 47 (37) | 50 (40) | 46 (39) |
|  | High Blood Pressure – n (%) | | 42 (11) | 41 (11) | 33 (9) |
|  | Diabetes – n (%) | | 16 (13) | 24 (19) | 22 (18) |
|  | History Heart Disease – n (%) | | 22 (17) | 22 (17) | 23 (19) |
|  | History Depression – n (%) | | 45 (35) | 42 (33) | 36 (30) |
| Fagerström Test for Nicotine Dependence | | | N=128 | N=127 | N=119 |
| Mild – n (%) | | | 19 (15) | 25 (20) | 21 (18) |
| Moderate – n (%) | | | 60 (47) | 57 (45) | 54 (45) |
| Severe – n (%) | | | 49 (38) | 45 (35) | 44 (37) |
| Beck Depression Inventory II | | | N=127 | N=127 | N=118 |
| Minimal – n (%) | | | 86 (68) | 92 (72) | 78 (66) |
| Mild – n (%) | | | 19 (15) | 19 (15) | 18 (15) |
| Moderate – n (%) | | | 15 (12) | 12 (9) | 14 (12) |
| Severe – n (%) | | | 7 (6) | 4 (3) | 8 (7) |
| Alcohol use per week – mean (SD) | | | 4 (6) | 4 (8) | 3 (5) |
| ^a^Participants were asked to select “White”, “Black”, or “Other, specify:”. Self-reported “Other” includes: Israeli, Indigenous, Asian, Pilipino, Urdu, Italian, Arab, Trinidadian, Moroccan, Nepalese, Spanish, Tunisian, East Indian.  ^b^Previously used abstinence aids includes: Varenicline, Bupropion, Nicotine Patch, Nicotine Gum, Nicotine Inhaler, Nicotine Lozenge, Nicotine QuickMist, Counseling, Other Aids (Acupuncture, Hypnosis, Laser, Apps)  ^c^Medical history was self-reported.  ^d^Other (Respiratory problems) includes: chronic pneumonia, shortness of breath, and sleep apnea  Abbreviations: IQR = interquartile range; SD = standard deviation, BMI = body mass index, Kg/m^2^ = kilogram divided by metre squared, cm = centimeter | | | | | |

**Table S5. Logistic regression results for lost-to-followup at week 12 beding the outcome, and baseline variables being the covariates.**

| **Variables** | **Estimate** | **Std Error** | **Prob>ChiSq** | **Lower 95%** | **Upper 95%** |
| --- | --- | --- | --- | --- | --- |
| Intercept | 2.71 | 3.09 | 0.38 | -3.27 | 8.96 |
| **Treatment[nicotine e-cigarettes plus counseling]** | -0.51 | 0.18 | 0.01 | -0.87 | -0.16 |
| Treatment[nonnicotine e-cigarettes plus counseling] | -0.12 | 0.17 | 0.49 | -0.46 | 0.22 |
| Age | -0.01 | 0.03 | 0.64 | -0.06 | 0.04 |
| Gender[Female] | -0.13 | 0.17 | 0.44 | -0.47 | 0.20 |
| Weight Baseline | -0.01 | 0.00 | 0.10 | -0.01 | 0.00 |
| Height | -1.08 | 1.87 | 0.56 | -4.87 | 2.53 |
| Education[No degree, diploma, or certification ] | 0.18 | 0.28 | 0.51 | -0.37 | 0.73 |
| Education[Completed secondary (high school) ] | 0.03 | 0.23 | 0.90 | -0.43 | 0.47 |
| Education[Some college/university ] | 0.03 | 0.19 | 0.88 | -0.35 | 0.40 |
| Cigarettes/day in the past 10 years | 0.00 | 0.01 | 0.88 | -0.02 | 0.03 |
| Years Smoked | 0.00 | 0.02 | 0.92 | -0.05 | 0.05 |
| Fagerström Test for Nicotine Dependence[1-0] | 0.35 | 0.37 | 0.34 | -0.36 | 1.09 |
| Fagerström Test for Nicotine Dependence[2-1] | 0.12 | 0.29 | 0.67 | -0.44 | 0.69 |
| Beck Depression Inventory II[Mild-Minimal] | -0.48 | 0.37 | 0.20 | -1.24 | 0.23 |
| Beck Depression Inventory II[Moderate-Mild] | 0.17 | 0.53 | 0.75 | -0.87 | 1.20 |
| Beck Depression Inventory II[Severe-Moderate] | -0.14 | 0.69 | 0.84 | -1.54 | 1.19 |
| Other Smoker(s) at Home[0] | -0.18 | 0.13 | 0.18 | -0.44 | 0.08 |
| High Cholesterol[0] | -0.04 | 0.16 | 0.82 | -0.35 | 0.28 |
| History Depression[0] | 0.07 | 0.15 | 0.64 | -0.22 | 0.36 |
| High Blood Pressure[0] | 0.14 | 0.17 | 0.39 | -0.18 | 0.47 |
| Respiratory Problems[0] | -0.16 | 0.14 | 0.25 | -0.44 | 0.12 |
| **History Heart Disease[0]** | -0.50 | 0.18 | 0.00 | -0.85 | -0.16 |
| Diabetes[0] | 0.15 | 0.20 | 0.44 | -0.23 | 0.55 |
| Alcohol use per week | -0.02 | 0.02 | 0.41 | -0.06 | 0.02 |
