# Supplementary material for: e-Cigarettes, Smoking Cessation, and Weight Change: Retrospective Secondary Analysis of the Evaluating the Efficacy of e-Cigarette Use for Smoking Cessation Trial
Source: JMIR Public Health Surveill. 2024 Sep 16;10:e58260. doi: 10.2196/58260 (PMC11443201; doi:10.2196/58260)
Supplement: Multimedia Appendix 2 [file publichealth_v10i1e58260_app2.docx]

**Multimedia Appendix 2. Imputation details**

The models used in the IPW-based causal inference methods (MSM and GPS) involve fitting linear or logistic regression models with complete data. Since the raw data contains a fair number of missing values, we impute them using two strategies. The first is imputation by prior knowledge, which includes: (i) missing values for e-cigarette related variables (used e-liquid cartridges returned, unused e-liquid cartridges returned, e-cigarettes used days per week, e-cigarette puffs used per week) in the counseling alone group which are set to 0; (ii) missing values for smoking abstinence at week 12 are set to 0, assuming these participants returned to smoking. For all remaining missing data, we perform imputation with XGBoost (22) for baseline variables and time-varying variables of interest utilizing all participants, including those who have censored weight at week 12. XGBoost, known for its robustness in handling complex datasets and capturing intricate patterns, potentially provides more accurate imputations than traditional methods, such as mean/median/mode imputation. However, practical implementation may be hindered by challenges such as the need for careful parameter tuning, susceptibility to overfitting, and increased computational resources. Unlike multiple imputation, XGBoost imputation directly learns patterns from data without relying on assumptions about missing data mechanisms or variable relationships. While XGBoost imputation's performance may vary based on data quality, quantity, and variable relationships, multiple imputation can yield robust estimates under specific assumptions, although it is sensitive to misspecification of the imputation model.

Baseline variables that are imputed include: *weight, height, average number of cigarettes smoked per day over the last 10 years, high cholesterol, Fagerström test for nicotine dependence score, and Beck depression inventory II (BDI) score. The following baseline variables were used for imputation: high cholesterol, BDI score, Fagerström test for nicotine dependence score, average number of cigarettes smoked per day over the last 10 years, height, age, gender, education, years smoked, other smoker at home, history depression, HBP, respiratory problems, history heart disease, diabetes, smoking abstinence, used abstinence aids, baseline BMI, baseline waist (inch), baseline systolic, baseline diastolic, baseline heart rate, used e-cigarettes before, tried to quit, Glover-Nilsson smoking behavioral questionnaire score, bypass, family heart disease, family high cholesterol, family HBP, family diabetes, history cancer, alcohol use, age start smoking, number of attempts to quit, chronic bronchitis, emphysema, more than one respiratory problems, country, first language, race, marital status, age of first cigarette, average cigarettes per day in the past year, COPD, asthma, heart attack, angina, stroke, angioplasty, baseline weight*.

For each variable to be imputed, a predictive model is set up in XGBoost with this variable as the response and all others as the predictors. Predicted values from the resulting model are then used to impute missing values.

Additionally, time varying variables are imputed, including *conventional cigarettes per week, on how many days did the participant smoke a conventional cigarette in the past week (even if only 1 puff), used e-liquid cartridges returned, unused e-liquid cartridges returned, and e-cigarette puffs used per week*. When imputing these variables, the baseline variables were used, along with the following time varying variables: *any smoking cessation aid, visit days, ECigeCigReduceNo, BRSAngry, BRSAnxious, BRSDepressed, BRSCraving, BRSConcentrating, BRSHungry, BRSInsomnia, BRSRestless, BRSImpatient, SAE, CounselingScript, Allocation Believed, Vital Status*.
