# Supplementary material for: e-Cigarettes, Smoking Cessation, and Weight Change: Retrospective Secondary Analysis of the Evaluating the Efficacy of e-Cigarette Use for Smoking Cessation Trial
Source: JMIR Public Health Surveill. 2024 Sep 16;10:e58260. doi: 10.2196/58260 (PMC11443201; doi:10.2196/58260)
Supplement: Multimedia Appendix 3 [file publichealth_v10i1e58260_app3.docx]

**Multimedia Appendix 3. Causal Statistical Modeling Details**

Since the distribution of each of the four variables is quite positively skewed, we log(1+x) transform each prior to model fitting. MSM and GPS with IPW are separately applied to the four preceding covariates. To comprehensively check for subgroup effects, we consider the following three subsets of the original dataset: i) Nicotine e-cigarettes plus counseling; ii) Nicotine e-cigarettes plus counseling and Nonnicotine e-cigarettes plus counseling; iii) Nonnicotine e-cigarettes plus counseling. The analysis workflow involves fitting (2 methods) x (4 covariates) x (3 subsets) = 24 models. We furthermore partition each covariate into 40 to 75 bins and refit each model with 1000 bootstrap iterations in order to obtain empirical bootstrap confidence limits, resulting in a grand total of 1,344,000 model fits.

**MA3.1 Doubly Robust Estimation (DRE) Analysis Steps**

1. Fit logistic regression model for censoring.
   1. Fit $logit\left( \Pr\left( Censor=1 \right) \right)\sim X_{baseline}$, and derive the predicted probabilities for censoring  $\hat{Pr}(Censor=1|X_{baseline})$.
   2. $X_{baseline}$ includes age, gender, education, years smoked conventional cigarettes, other smoker(s) at home, history depression, HBP, respiratory problems, history heart disease, diabetes, baseline weight, height, cigarettes/day in the past 10 years, Fagerström Test for Nicotine Dependence score, Beck Depression Inventory II score, high cholesterol, smoking abstinence, alcohol use, any smoking cessation aid. And the quadratic terms of the continuous variables age, cigarettes/day in the past 10 years, baseline weight, years smoked conventional cigarettes, height, alcohol use.
   3. Fit $logit\left( \Pr\left( Censor=1 \right) \right)\sim1$, and derive the predicted probabilities for censoring  $\hat{Pr}(Censor=1)$.
   4. Calculate the stabilized censoring weights: $SW_{censor}=\hat{Pr}(Censor=1)/\hat{Pr}(Censor=1|X_{baseline})$.
2. Fit logistic regression for the assigned treatment to accommodate for the selection bias.
   1. Fitted $logit\left( \Pr\left( Trt=Trt_{i} \right) \right)\sim X_{baseline}, i=1, 2, 3$. Derive the predicted probabilities for each treatment arm $Pr(Trt=Trt_{i}|X_{baseline})$.
   2. $Trt_{1}=Nicotine e-cigarettes; Trt_{2}=Nonnicotine e-cigarettes;Trt_{3}=Counseling only$.
   3. Fit $logit\left( \Pr\left( Trt=Trt_{i} \right) \right)\sim1, i=1, 2, 3$. Derive the predicted probabilities for each treatment arm $Pr(Trt=Trt_{i})$.
   4. Calculated the stabilized treatment weights: $SW_{trt}=\Pr\left( Trt=Trt_{i} \right)/Pr(Trt=Trt_{i}|X_{baseline}), i=1, 2, 3$.
3. Calculate the overall weight for the downstream linear regression models: for the ITT analysis, $W=SW_{censor}$; for the as-treated analysis: $W=SW_{censor}\times SW_{trt}$.
4. Fit Standardization via the parametric g-formula (27)
   1. Make three copies of the data, and separately replace all the treatment variable value to one of the treatment arms for all the subjects.
   2. Set the outcome variable weight gain ($Y$) to missing for these three datasets.
   3. Fit the linear model for weight gain: $Y\sim X_{baseline}$, and derive the predicted weight gain values and formula $\hat{Y}$. Apply the prediction formulas to the three copies of datasets and derive $\hat{Y}^{Trt_{i}}$, which is the counterfactually predicted values under treatment $i$.
5. Calculate the doubly robust estimator
   1. $\hat{DRE}_{Trt_{i}}=\frac{1}{n}\sum_{i=1}^{n} \left( I\left( Trt=Trt_{i} \right)\times\frac{Y_{i}-{\hat{Y_{i}}}^{Trt_{i}}}{W_{i}}+{\hat{Y_{i}}}^{Trt_{i}} \right)$
6. Use bootstrapping to derive the confidence intervals for the DREs.

**MA3.2 Marginal Structural Model (MSM) Analysis Steps**

1. Fit Logistic regression model for censoring.
   1. Fit $logit\left( \Pr\left( Censor=1 \right) \right)\sim X_{baseline}$, and derived the predicted probabilities for censoring  $\hat{Pr}(Censor=1|X_{baseline})$.
   2. $X_{baseline}$ includes age, gender, education, years smoked conventional cigarettes, other smoker(s) at home, history depression, HBP, respiratory problems, history heart disease, diabetes, baseline weight, height, cigarettes/day in the past 10 years, Fagerström Test for Nicotine Dependence score, Beck Depression Inventory II score, high cholesterol, smoking abstinence, alcohol use, any smoking cessation aid.
   3. Fit $logit\left( \Pr\left( Censor=1 \right) \right)\sim1$, and derive the predicted probabilities for censoring  $\hat{Pr}(Censor=1)$.
   4. Calculate the stabilized censoring weights: $SW_{censor}=\hat{Pr}(Censor=1)/\hat{Pr}(Censor=1|X_{baseline})$.
2. For each log1p-transformed continuous treatment variable $Z$,
   1. Fit the linear regression model: $Z_{j}\sim X_{baseline}+Z_{j^{'}\neq j}, j=1,2,3,4. Z_{1}=$ Log(1+ conventional cigarettes per week); $Z_{2}=$ Log(1+ e-cigarette puffs used per week); $Z_{3}=$ Log(1+ used e-liquid cartridges returned); $Z_{4}=$ Log(1+ unused e-liquid cartridges returned).
   2. Derive the predicted treatment values along with the residuals. Compute the standard deviation of the residuals and further derive the corresponding empirical Normal density function $f\left( Z_{j}|X_{baseline},Z_{j^{'}\neq j} \right)$.
   3. Fit the linear regression model: $Z_{j}\sim1$. Similarly, derive the residuals and the corresponding Normal density function $f\left( Z_{j} \right)$.
   4. Derive the stabilized weights for treatment $Z_{j}$: $W_{Z_{j}}= f\left( Z_{j} \right)/f\left( Z_{j}|X_{baseline},Z_{j^{'}\neq j} \right)$.
   5. Extreme stabilized weights were truncated at their 97.5 percentile.
3. Calculate the combined weight for the downstream MSM analysis by $W_{Z_{j}}\times SW_{censor}$. Thus, we adjusted for censoring effect and the selection bias at the same time.
4. Perform the MSM analysis using each of the treatment variable separately:
   1. Fit weighted linear regression model for weight gain ($Y$): $Y\sim Z_{j}, j=1, 2, 3, 4$, where the regression weight is specified as $W_{Z_{j}}\times SW_{censor}.$
   2. Save the predicted mean weight gain for various $Z_{j}$ values.
5. Use bootstrapping to derive the 95% Cis for the average weight gain at each treatment level.

**MA3.3 Generalized Propensity Scoring (GPS) Analysis Steps**

1. Similar to the MSM analysis, we derive the stabilized weight for censoring $SW_{censor}$.
2. The GPS for each continuous treatment score is calculated as follows:
   1. Fit the linear regression model: $Z_{j}\sim X_{baseline}+Z_{j^{'}\neq j}, j=1,2,3,4. Z_{1}=$ Log(1+ conventional cigarettes per week); $Z_{2}=$ Log(1+ e-cigarette puffs used per week); $Z_{3}=$ Log(1+ used e-liquid cartridges returned); $Z_{4}=$ Log(1+ unused e-liquid cartridges returned).
   2. Derive the predicted treatment values along with the residuals. Compute the standard deviation of the residuals and further derive the corresponding empirical Normal density function $f\left( Z_{j}|X_{baseline},Z_{j^{'}\neq j} \right)$.
   3. Fit weighted linear regression model: $Y\sim Z_{j}+f\left( Z_{j}|X_{baseline},Z_{j^{'}\neq j} \right)+f\left( Z_{j}|X_{baseline},Z_{j^{'}\neq j} \right)\times Z_{j}, j=1, 2, 3, 4,$ where the regression weight is set to $SW_{censor}$. Save the estimated parameter values.
   4. Set a fixed value $\tilde{Z}_{j}$ to $Z_{j}$ for all the subjects and recalculate the GPS values $f\left( \tilde{Z}_{j}|X_{baseline},Z_{j^{'}\neq j} \right)$. Use the estimated parameters and the current GPS values to calculate the mean weight gain. Vary $\tilde{Z}_{j}$ values, and repeat this procedure.
3. Use bootstrapping to derive the 95% CIs for the average weight gain at each treatment level.
