# Supplementary material for: e-Cigarettes, Smoking Cessation, and Weight Change: Retrospective Secondary Analysis of the Evaluating the Efficacy of e-Cigarette Use for Smoking Cessation Trial
Source: JMIR Public Health Surveill. 2024 Sep 16;10:e58260. doi: 10.2196/58260 (PMC11443201; doi:10.2196/58260)
Supplement: Multimedia Appendix 4 [file publichealth_v10i1e58260_app4.docx]

**Multimedia Appendix 4. Supplementary Figure**

**Figure S1. CONSORT-flow diagram showing the attrition by visits.**
