# Supplementary material for: e-Cigarettes, Smoking Cessation, and Weight Change: Retrospective Secondary Analysis of the Evaluating the Efficacy of e-Cigarette Use for Smoking Cessation Trial
Source: JMIR Public Health Surveill. 2024 Sep 16;10:e58260. doi: 10.2196/58260 (PMC11443201; doi:10.2196/58260)

**Multimedia Appendix 5. Power Analysis**

First, we describe some terms used in this power analysis:

- Power = 1 - β = 0.80 (80% power, which implies a 20% Type II error rate).
- Sample size (n) = 376, 257, 192, 101, 91 (assuming this is the total sample size).
- Significance level (α) = 0.05 (5% significance level).
- Standard deviation (σ) = need to know or estimate the standard deviation of the outcome variable in the study, or from previous studies.
- Distribution of Baseline weight in kg:

|  | **Nicotine e-cigarettes plus counseling** | **Nonnicotine e-cigarettes plus counseling** | **Counseling alone** |
| --- | --- | --- | --- |
| Mean | 82.44 | 81.46 | 79.67 |
| Std Dev | 20.47 | 17.88 | 16.61 |

- Calculation of the average treatment effect (ATE):

$$power=1-\beta= \Phi\left( -Z_{1-\frac{\alpha}{2}}+\frac{\Delta}{\sqrt{\frac{\sigma_{1}^{2}}{n_{1}}+\frac{\sigma_{2}^{2}}{n_{2}}}} \right)$$

In JMP, we employ the DOE platform to perform the power analysis. From DOE > Sample Size Explorers > Power > Power for Two Independent Sample Means, we derive the following report.


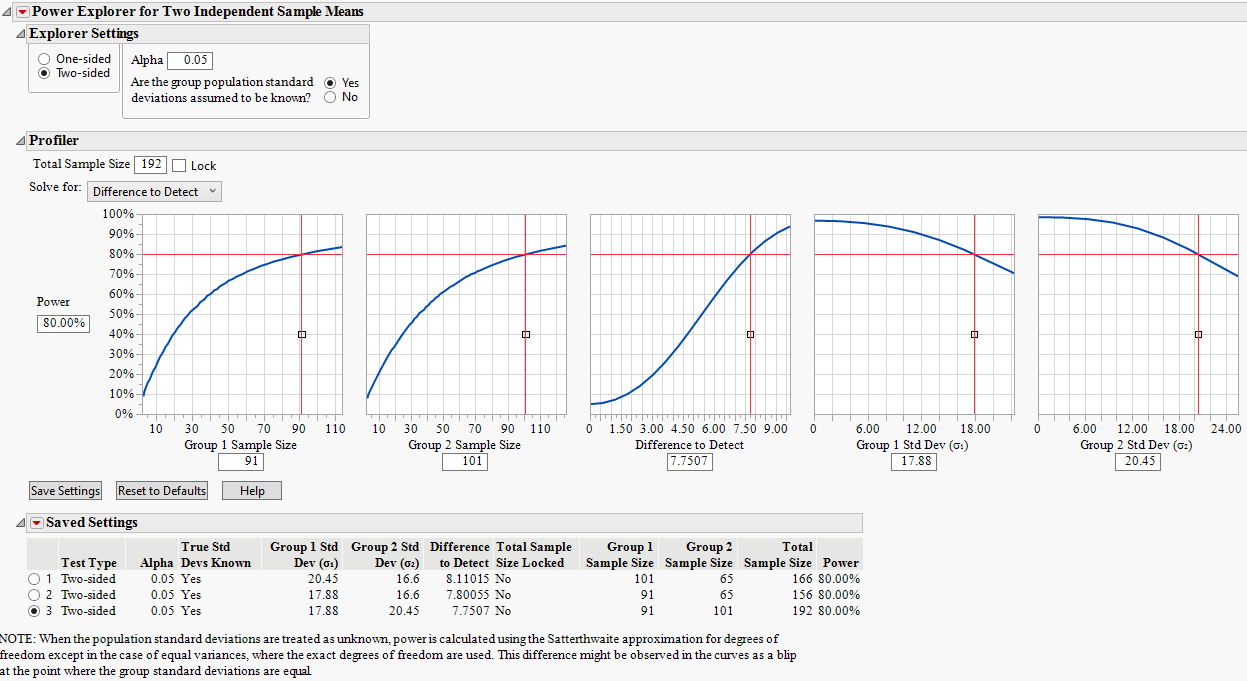


The effect size needs to be around 7-8 kg or larger to be detected in the case of this paper.

Additionally, when comparing two arms, if we assume that we have an equal number of enrollments in each group, with a standard deviation of 18, and alpha=0.05, for an effect size of 5kg, we will need N1 = N2 = 204 patients in each arm to achieve 80% power.

To achieve 80% power, with sd=18, we calculate the sample size needed when varying the effect size in a two-arm setting. Here, sample size means N1+N2 = 2*N1 – the scenario when we have two groups and the number of patients in each group is the sample (sample size ratio = 0.5).


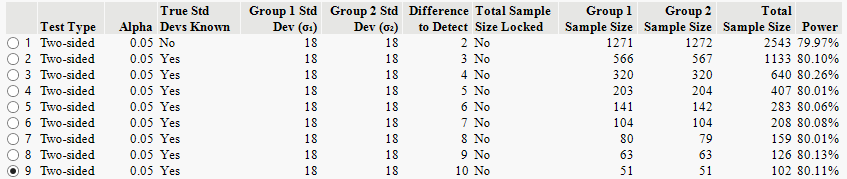


Our three pairwise comparisons result in a sample size ratio of 0.53, 0.58, and 0.61. The estimated two groups' total sample size is similar across these ratio values.


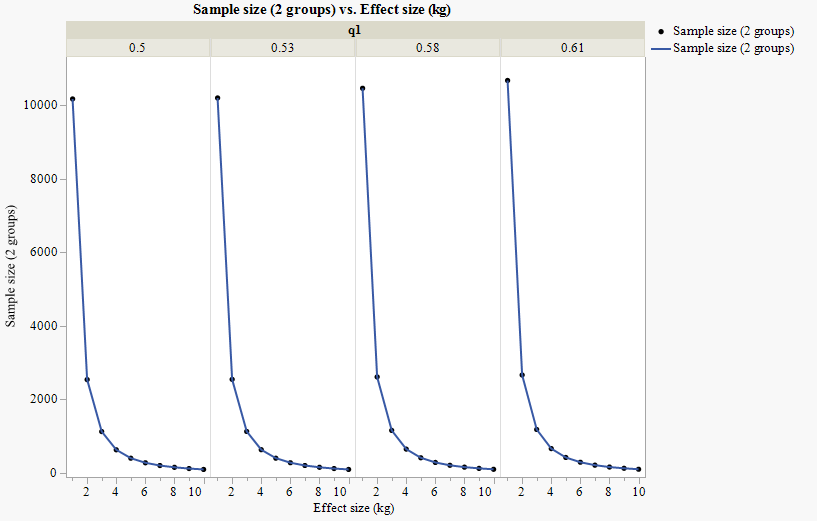

Supplement: Multimedia Appendix 5 [file publichealth_v10i1e58260_app5.docx]
