# Supplementary material for: e-Cigarettes, Smoking Cessation, and Weight Change: Retrospective Secondary Analysis of the Evaluating the Efficacy of e-Cigarette Use for Smoking Cessation Trial
Source: JMIR Public Health Surveill. 2024 Sep 16;10:e58260. doi: 10.2196/58260 (PMC11443201; doi:10.2196/58260)
Supplement: Multimedia Appendix 6 [file publichealth_v10i1e58260_app6.docx]

**Multimedia Appendix 6. Continuous Abstinence Rate (CAR) analysis**

The distribution of CAR across each visit and treatment arm is as follows: The nicotine e-cigarettes plus Counseling group consistently exhibits the highest CAR throughout all visits, while the counseling alone group consistently demonstrates the lowest CAR across all visits.

We created the following figure as a descriptive graph for mean weight change at each visit vs. CAR. Without adjusting for other potential confounding variables, we can observe that in the nicotine e-cigarettes plus Counseling group, as the CAR value decreases over time, the average weight gain increases.
